# Integrative evidence reveals a new species of Hysterothylacium (Nematoda: Ascaridoidea), with the characterization of its complete mitochondrial genome

**DOI:** 10.1016/j.ijppaw.2025.101042

**Published:** 2025-02-02

**Authors:** Hui-Xia Chen, Hai-Xia Qiao, Wen-Ting Zhao, Xiao-Hong Gu, Liang Li

**Affiliations:** a Hebei Collaborative Innovation Center for Eco‐Environment, Hebei Key Laboratory of Animal Physiology, Biochemistry and Molecular Biology, College of Life Sciences, Hebei Normal University, 050024, Shijiazhuang, Hebei Province, PR China; b Ministry of Education Key Laboratory of Molecular and Cellular Biology, 050024, Shijiazhuang, Hebei Province, PR China

**Keywords:** Parasite, Raphidascarididae, Integrative taxonomy, Species delimitation, ASAP, Marine fish

## Abstract

The genus *Hysterothylacium* (Ascaridida: Raphidascarididae) is among the commonest groups of parasitic nematodes occurring in the digestive tract of marine fishes. In the present study, a new species of *Hysterothylacium*, *H. hainanense* sp. n. collected from *Uranoscopus tosae* (Jordan & Hubbs) and *U. japonicus* Houttuyn (Perciformes: Uranoscopidae) in the Chinese waters was described using integrative methods, including light and scanning electron microscopy, and ASAP and BI analyses based on the ITS sequence data. The complete mitochondrial genome of the new species was sequenced and annotated, which represents the first mitogenomic data for the genus *Hysterothylacium*, and also for the family Raphidascarididae. The mitogenome of *H. hainanense* sp. n. is 14059 bp in length, including 12 protein coding genes (missing *atp*8), 22 tRNA genes, 2 rRNA genes and 2 non-coding regions, which has 67.0% of overall A + T content, and represents the lowest level of A + T content in the ascaridoid mitogenomes reported so far. Molecular phylogenetic results suggested a close affinity between *H. hainanense* sp. n. and *H. fabri* in the genus *Hysterothylacium*.

## Highlights

• *Hysterothylacium hainanense* sp. n. was described based on integrative evidence.
• BI analysis suggested a close affinity between *H. hainanense* sp. n. and *H. fabri* in *Hysterothylacium.*
• The mitogenome of *H. hainanense* sp. n. representing the first mitogenomic data for the Raphidascarididae, was presented.

## Graphical abstract

**Figure:**
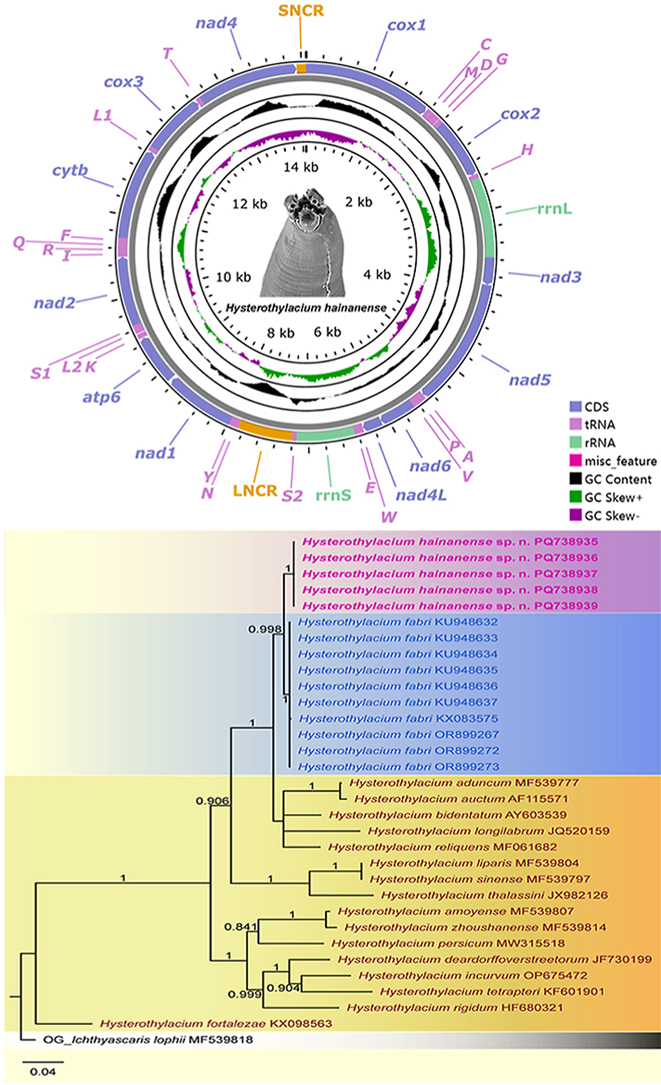

## 1 Introduction

The family Raphidascarididae is a large group of ascaridoid nematodes, with over 200 species mainly parasitic in teleosts (Bruce et al., 1994; Li et al., 2018). In the Raphidascarididae, the genus *Hysterothylacium* (Ascaridida: Raphidascarididae) is among the commonest groups of parasitic nematodes occurring in the digestive tract of marine fishes (Deardorff and Overstreet, 1980; Bruce et al., 1994; Li et al., 2007, 2012a, 2012b; Moravec and Justine, 2015; Shamsi et al., 2016; Chen et al., 2018). To date, more than 70 nominal species of *Hysterothylacium* have been reported from various fish hosts worldwide (Bruce et al., 1994; Li et al., 2007; Moravec and Justine, 2015; Shamsi, 2017). Infection of *Hysterothylacium* nematodes can affect the growth rate and health of the fish hosts, and even results in mortalities for fish fries (Balbuena et al., 2000). Additionally, some *Hysterothylacium* species seem to be the etiological agent for human anisakidosis (Yagi et al., 1996).

In recent years, the integrative approaches based on the morphological and molecular data, were widely used in the taxonomy of *Hysterothylacium*, especially for the discovery of cryptic species, evaluation of morphological variability, and species identification of different developmental stages of parasites (Li et al., 2012a, 2012b, 2017; Zhao et al., 2017; Chen et al., 2018; Zhang et al., 2018; Guo et al., 2020; De Benedetto et al., 2022; Gelen and Pekmezci, 2023; Morales-Yuste et al., 2024). However, the current genetic data of *Hysterothylacium* spp. remains insufficient. To date, approximately 70% of *Hysterothylacium* species have not been genetically sequenced, and no data on the mitochondrial genome of *Hysterothylacium* or raphidascaridid nematodes have been reported so far.

In the present study, a new species of *Hysterothylacium* was described using both light and scanning electron microscopy, based on specimens collected from *Uranoscopus tosae* (Jordan & Hubbs) and *U. japonicus* Houttuyn (Perciformes: Uranoscopidae) in the Chinese waters. In order to test the validity and clarify the phylogenetic relationships between the new species and its congeners, Assemble Species by Automatic Partitioning (ASAP) analyses and Bayesian inference (BI) were performed based on the internal transcribed spacer (ITS) rDNA sequence data. Furthermore, the complete mitochondrial genome of the new species was sequenced and annotated to determine the pattern of mitogenomic evolution of the genus *Hysterothylacium* and the family Raphidascarididae.

## 2 Materials and methods

### 2.1 Morphological study

A total of two individuals of *U*. *tosae* and two individuals of *U*. *japonicus* caught by the local fishermen from off Sanya, Hainan Island, China, were examined for parasites. Nematode specimens collected from the intestine and stomach of the two fish hosts were washed using physiological saline, then fixed and stored in 80% ethanol. For light microscopy, nspecimens were cleared in glycerin. Drawings were made with the aid of Nikon microscope drawing attachment. For scanning electron microscopy (SEM), the anterior and posterior ends of specimens were transferred to 4% formaldehyde solution, then post-fixed in 1% OsO₄, dehydrated via an ethanol series and acetone and critical point dried. The specimens were coated with gold and examined using a Hitachi S-4800 scanning electron microscope (Hitachi Ltd., Tokyo, Japan) at an accelerating voltage of 20 kV. Type specimens were deposited in College of Life Sciences, Hebei Normal University, Hebei Province, China. Measurements (the range, followed by the mean in parentheses) were given in micrometres (μm) unless otherwise stated.

### 2.2 Molecular procedures

The mid-body of two male and two female nematode individuals from *U*. *tosae*, and one female individual from *U*. *japonicus* was used for molecular analysis. Genomic DNA was extracted using a Column Genomic DNA Isolation Kit (Shanghai Sangon, China) according to the manufacturer's instructions. DNA was eluted in elution buffer and kept at *-*20°C until use. The used primers and cycling conditions for amplifying the ITS target region, and the procedures for sequencing and analysing, were according to the previous study (Li et al., 2018). All of the ITS sequences obtained herein were deposited in the GenBank database (http://www.ncbi.nlm.nih.gov, PQ738935‒PQ738939).

### 2.3 ASAP and BI analyses

The ASAP (Puillandre et al., 2021) was executed using the ASAP online server (https://bioin fo.mnhn.fr/abi/public/asap) under the Kimura (K80) ts/tv model based on the ITS sequence data. The result of ASAP with lowest score was considered as the optimal group number in the present study. Bayesian inference was performed using MrBayes 3.2.7 (Ronquist et al., 2012), with two parallel runs (5 000 000 generations) under the optimal model HKY + F + G4. *Ichthyascaris lophii* Wu, 1949 (Ascaridida: Raphidascarididae) was chosen as the out-group for both ASAP and BI analyses. The detailed information of *Hysterothylacium* species included in the ASAP and BI analyses was provided in Table 1.

**Table 1:** Detailed information of *Hysterothylacium* species with their ITS genetic data included in the ASAP and BI analyses.

Species	Host	Locality	GenBank ID	References
**Ingroup**				
*H*. *aduncum*	*Conger myriaster*	China	MF539777	Chen et al. (2018)
*H*. *amoyense*	*Conger myriaster*	China	MF539807	Chen et al. (2018)
*H*. *auctum*	*Zoarces viviparus*	Baltic Sea	AF115571	Szostakowska et al. (2001)
*H*. *bidentatum*	*—*	–	AY603539	Unpublished
*H*. *deardorffoverstreetorum*	*Paralichthys isosceles*	Brazil	JF730199	Knoff et al. (2012)
*H. fabri*	*Mullus surmuletus*	Italy	KU948632‒KU948637	Tedesco et al. (2018)
	*Trigla lyra*	Italy	KX083575	Costa et al. (2018)
	*Mullus surmuletus*	Spain	OR899267	Morales-Yuste et al. (2024)
	*Mullus barbatus*	Spain	OR899272	Morales-Yuste et al. (2024)
	*Citharus linguatula*	Spain	OR899273	Morales-Yuste et al. (2024)
*H*. *fortalezae*	*Maurolicus weitzmani*	USA	KX098563	Andres et al. (2016)
*H. hainanense* sp. n.	*Uranoscopus tosae*	China	PQ738935‒ PQ738938	Present study
	*Uranoscopus japonicus*	China	PQ738939	Present study
*H. incurvum*	*Xiphias gladius*	Atlantic Ocean	OP675472	De Benedetto et al. (2022)
*H*. *liparis*	*Conger myriaster*	China	MF539804	Chen et al. (2018)
*H*. *longilabrum*	*Siganus fuscescens*	China	JQ520159	Li et al. (2012a)
*H*. *persicum*	*Scomber australasicus*	Australia	MW315518	Unpublished
*H*. *reliquens*	*Brachirus orientalis*	Iraq	MF061682	Li et al. (2018)
*H*. *rigidum*	*Lophius piscatorius*	Ireland	HF680321	Unpublished
*H*. *sinense*	*Conger myriaster*	China	MF539797	Chen et al. (2018)
*H*. *tetrapteri*	*Kajikia audax*	China	KF601901	Unpublished
*H*. *thalassini*	*Priacanthus tayenus*	China	JX982126	Liu et al. (2013)
*H*. *zhoushanense*	*Conger myriaster*	China	MF539814	Chen et al. (2018)
**Outgroup**				
*Ichthyascaris lophii*	*Conger myriaster*	China	MF539818	Chen et al. (2018)

### 2.4 Mitochondrial genome sequencing, assembly and annotation

A total of 30 Gb clean genomic data were generated based on one female individual, using the Pair-End 150 sequencing method on the Illumina NovaSeq 6000 platform by Novogene (Tianjin, China). The complete mitochondrial genome was assembled and annotated using GetOrganelle v1.7.2a (Jin et al., 2020), MitoS web server (http://mitos2.bioinf.uni-leipzig.de/index.py), MitoZ v2.4 (Meng et al., 2019), ORF finder (https://www.ncbi.nlm.nih.gov/orffinder/), ViennaRNA package (Gruber et al., 2015), MitoS2 (Bernt et al., 2007) and RNAstructure v6.3 (Reuter and Mathews, 2010). The CGView online server V1.0 (http://stothard.afns.ualberta.ca/cgview_server/) was employed to visualize and depict gene element features. The base composition, amino acid usage and relative synonymous codon usage (RSCU) were calculated by Python script, which refers to Codon Adaptation Index (CAI) (Cock et al., 2009). The complete mitochondrial genome of this new species obtained was deposited in the NCBI database (http://www.ncbi.nlm.nih.gov, PQ740960).

## 3 Results

### **3.1** Description of *Hysterothylacium hainanense* sp. n (Fig. 1, Fig. 2)

**Fig. 1:**
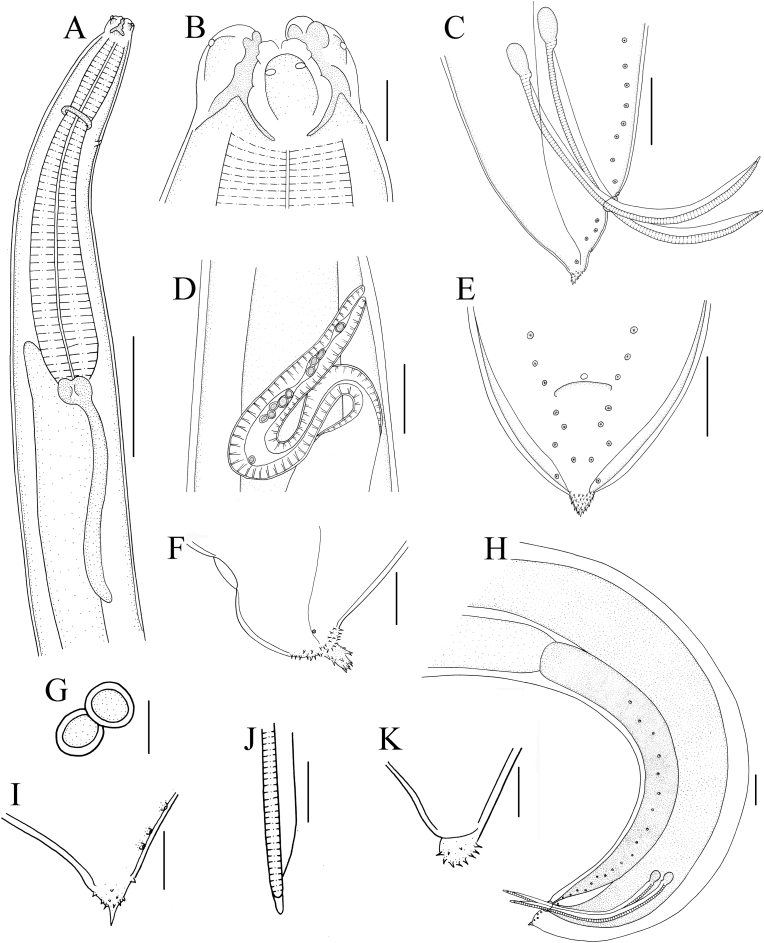
*Hysterothylacium hainanense* sp. n. collected from *Uranoscopus tosae* (Jordan & Hubbs) in China. A: anterior part of male, lateral view; B: cephalic extremity of male, dorsal view; C: tail of male (showing spicules), lateral view; D: region of vulva; lateral view; E: tail of male, ventral view; F: tail tip of female, lateral view; G: eggs; H: posterior end of male (showing ejaculatory duct, caudal papillae and spicules), lateral view; I, K: tail tip of male; J: tip of spicule, lateral view. *Scale-bars*: A = 500 μm; B, F, G = 50 μm; C, E, H = 100 μm; D = 200 μm; I‒K = 30 μm.

**Fig. 2:**
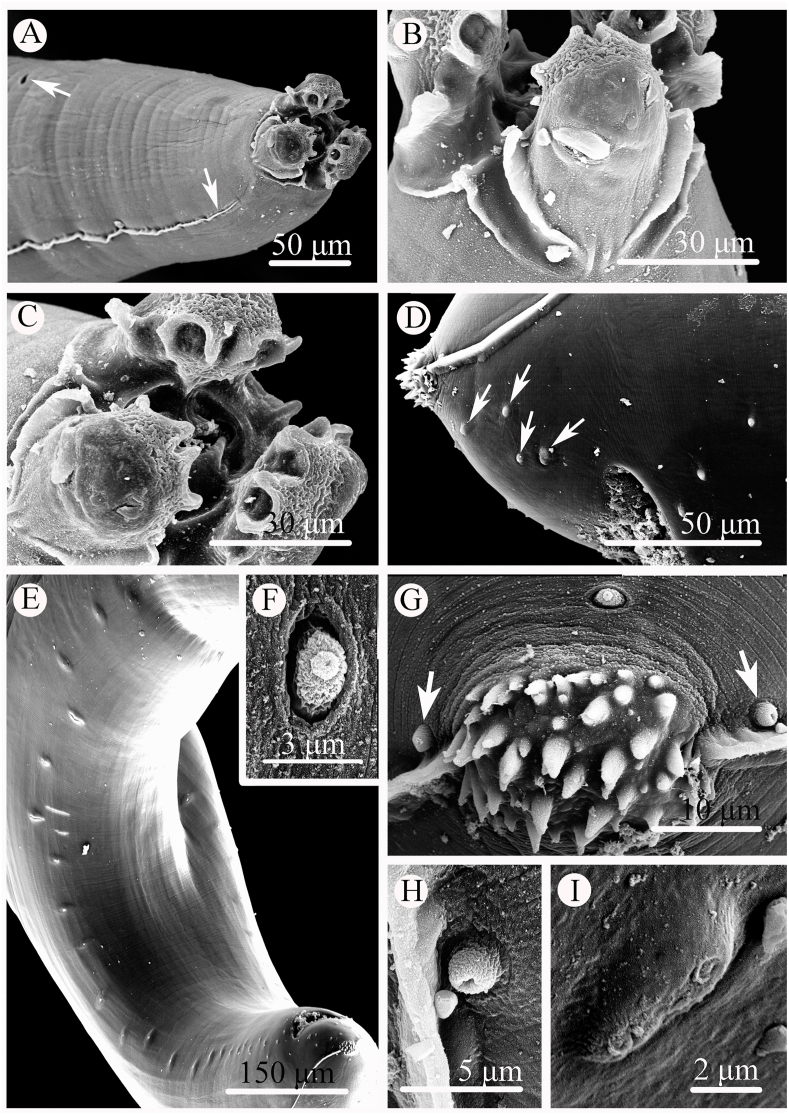
Scanning electron micrographs of *Hysterothylacium hainanense* sp. n. collected from *Uranoscopus tosae* (Jordan & Hubbs) in China. A: anterior part of male (lateral alae and excretory pore arrowed), ventro-lateral view; B: cephalic extremity of male, ventro-lateral view; C: cephalic extremity of male, apical view; D: caudal region of male (postcloacal papillae arrowed), lateral view; E: posterior end of male, subventral view; F: magnified image of postcloacal papilla; G: magnified image of tail tip of male (phasmids arrowed); H: magnified image of phasmid; I: magnified image of postcloacal double papilla.

Small to medium sized, whitish nematodes with finely transversely striated cuticle. Body cylindrical, maximum width at about region of mid-body. Cephalic extremity with 3 lips, almost equal in size, each lip with deep postlabial grooves and prominent lateral flanges (Fig. 1, Fig. 2A, B). Dorsal lip with one pair of large double cephalic papillae; ventro-lateral lips each with single double cephalic papilla, small papilla and amphid (Fig. 1, Fig. 2A‒C). Proximal region of each lip divided into 4 lobes (Fig. 2A‒C). Interlabia small, triangular, about 1/4–1/3 length of lips (Fig. 1, Fig. 2A‒C). Lateral alae narrow, extending from some distance from base of ventro-lateral lips to base of tail tip (Fig. 2A–D). Oesophagus muscular, distinctly broader posteriorly than anteriorly (Fig. 1A). Deirids not observed. Nerve ring at about 1/3 of oesophageal length. Excretory pore just posterior to nerve ring (Fig. 1A). Ventriculus oval, distinctly narrower than posterior region of oesophagus. Intestinal caecum short, about 1/5 of oesophageal length (Fig. 1A). Ventricular appendix distinctly longer than intestinal caecum (Fig. 1A). Tail of both sexes conical, tip covered by numbers of small spines (Fig. 1, Fig. 2D, G).

***Male*** (based on 6 mature specimens): Body 13.8–28.0 (20.0) mm long; maximum width 487–1440 (744). Lips 55–78 (68.9) long, 45–100 (66.9) wide. Interlabia 13–33 (24.1) long. Oesophagus 1.02–1.73 (1.28) mm long, representing 4.70–10.8 (6.80) % of body length. Intestinal caecum 195–317 (261) long, representing 17.3–26.5 (22.4) % of oesophageal length. Ventriculus 68–146 (110) long, 98–185 (134) wide. Ventricular appendix 913–1019 (959) long, representing 63.4–100 (83.8) % of oesophageal length; ratio of intestinal caecum to ventricular appendix 1: 3.0–5.0 (1: 3.5). Nerve-ring and excretory pore 400–652 (484) and 478–798 (574) from cephalic extremity, respectively. Ejaculatory duct 1.37–2.14 (1.86) mm long. Spicules alate, pointed at end, equal in length, 374–795 (555) long, representing 24.2–38.1 (32.5) % of ejaculatory duct length and 2.30–3.20 (2.80) % of body length, respectively (Fig. 1C–H, G). Caudal papillae 30–33 pairs in total, arranged as following: precloacal 25–27 pairs, paracloacal absent and postcloacal 4–5 pairs (1st pair being double) (Fig. 1, Fig. 2). Single medio-ventral precloacal papilla present. Tail 98–292 (172) long. Lateral phasmids located at base of tail tip (Fig. 2G and H).

***Female*** (based on 8 mature specimens): Body 13.7–49.0 (31.1) mm long; maximum width 485–1214 (894). Lips 93–123 (106) long, 83–132 (106) wide. Interlabia 12–50 (37.4) long. Oesophagus 1.07–1.66 (1.35) mm long, representing 3.10–7.80 (4.70) % of body length. Intestinal caecum 194–439 (278) mm long, representing 15.2–29.0 (20.6) % of oesophageal length. Ventriculus 88–184 (135) long, 112–214 (164) wide. Ventricular appendix 586–1262 (945) long, representing 48.6–100 (70.7) % of oesophageal length; ratio of intestinal caecum to ventricular appendix 1: 2.3–5.0 (1: 3.5). Nerve-ring and excretory pore 303–537 (469) and 342–621 (535) from cephalic extremity, respectively. Vulva slit-like, without swollen lips, pre-equatorial, 7.69–14.8 (11.1) mm from cephalic extremity, representing 20.8–42.4 (31.6) % of body length (Fig. 1D). Vagina muscular, uterus didelphic. Eggs almost rounded or ovoid, 40–50 (46) × 45–60 (53) (n = 20) (Fig. 1G). Tail 126–346 (220) long. Lateral phasmids present (Fig. 1F).

*Type host*: *Uranoscopus tosae* (Jordan & Hubbs) (Perciformes: Uranoscopidae).

*Other host*: *Uranoscopus japonicus* Houttuyn (Perciformes: Uranoscopidae).

*Type locality*: Off Sanya, Hainan Island, China.

*Site of infection*: Intestine and stomach.

*Prevalence and intensity*: 2 out of 2 individuals of *U*. *tosae* infected with intensity 1–7 (mean 4.0) nematodes per fish; 2 out of 2 individuals of *U*. *japonicus* infected with intensity 1–6 (mean 3.5) nematodes per fish.

*Type specimens*: Holotype:1 male (HBNU–N–F20241220CL), allotype: 1 female (HBNU–N–F20241121CL), paratypes: 4 males, 2 females (HBNU–N–F20241122CL) collected from *U*. *tosae*; paratypes: 2 males, 5 females (HBNU–N–F20241122CL) collected from *U. japonicus*; all type specimens deposited in College of Life Sciences, Hebei Normal University, Hebei Province, China.

*Etymology*: The species name refers to the type locality (Hainan Island).

### 3.2 Species delimitation of *Hysterothylacium* spp

**Fig. 3:**
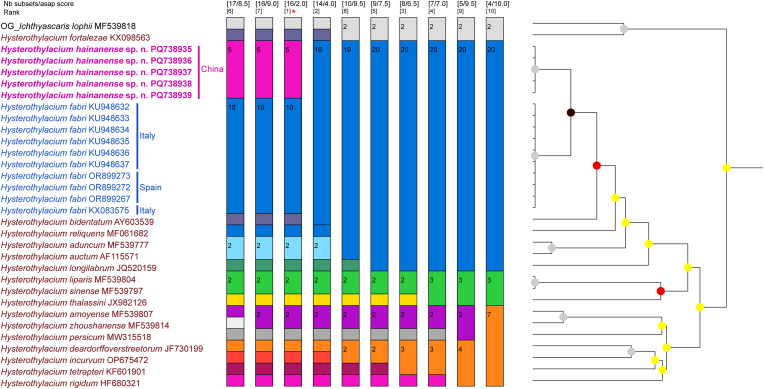
Assemble species by automatic partitioning (ASAP) analysis of *Hysterothylacium* spp. based on the ITS sequence data. *Ichthyascaris lophii* (Ascaridida: Raphidascarididae) was chosen as the out-group. Asterisk indicated the optimal result recommended by ASAP.

**Fig. 4:**
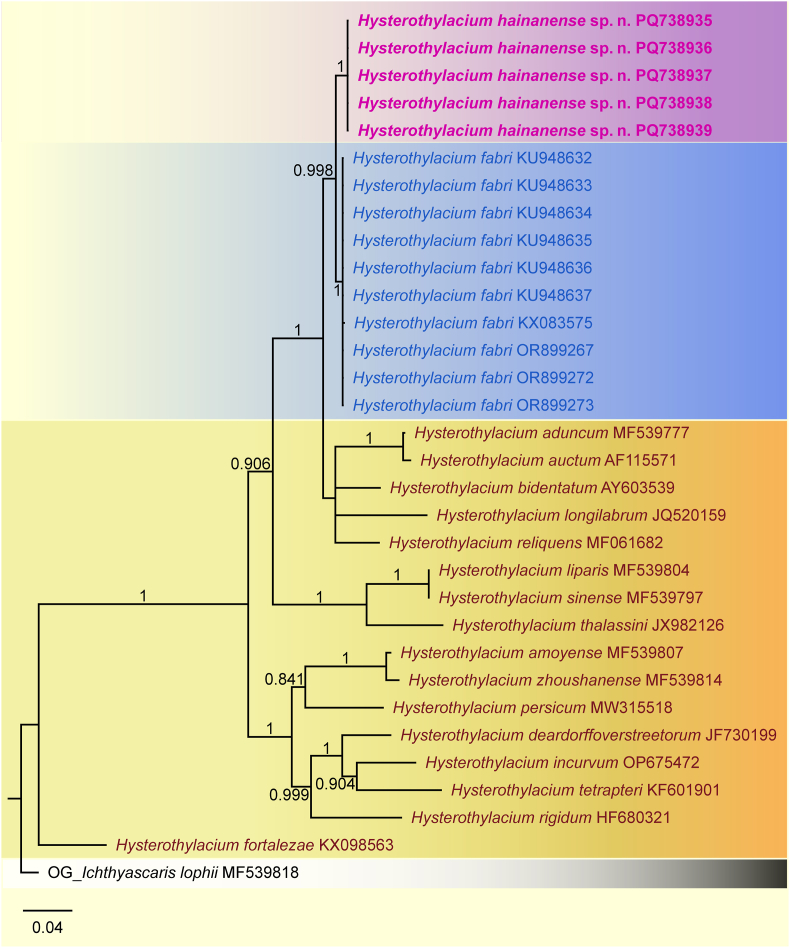
Bayesian inference (BI) based on the ITS sequence data showing the phylogenetic relationships of *Hysterothylacium* spp. *Ichthyascaris lophii* (Ascaridida: Raphidascarididae) was chosen as the out-group. Bayesian posterior probabilities (BPP) ≥0.80 in BI tree were shown.

Five ITS sequences of *H. hainanense* sp. n. from two fish hosts obtained here are all 1002 bp, with no nucleotide divergence detected. The results of pairwise comparison of the partial ITS sequences of *H. hainanense* sp. n. and its closely related species *H. fabri* were provided in Table 2. The ASAP result displayed *H. hainanense* sp. n. representing a separate species from *H. fabri* (Fig. 3), but did not support the current species partition of these species *H. aduncum* and *H. auctum*, *H. liparis* and *H. sinense*, and *H. amoyense* and *H. zhoushanense*. The phylogenetic result based on the ITS sequence data using BI method also showed *H. hainanense* sp. n. and *H. fabri* represent two distinct genetic lineages (Fig. 4), which was concordant with the ASAP result.

**Table 2:** Nucleotide polymorphisms in the partial ITS region between *Hysterothylacium hainanense* sp. n. and *H*. *fabri*.

GenBank ID	Sequence polymorphisms revealed at alignment positions													
	28	58	62	245	256	367	557	590	609	624	709	736	815	853
***Hysterothylacium hainanense* sp. n.**														
PQ738935	G	T	C	G	C	A	A	T	T	C	G	A	T	G
PQ738936	G	T	C	G	C	A	A	T	T	C	G	A	T	G
PQ738937	G	T	C	G	C	A	A	T	T	C	G	A	T	G
PQ738938	G	T	C	G	C	A	A	T	T	C	G	A	T	G
PQ738939	G	T	C	G	C	A	A	T	T	C	G	A	T	G
***Hysterothylacium fabri***														
KU948632	A	G	T	A	T	T	–	C	A	–	A	T	C	A
KU948633	A	G	T	A	T	T	–	C	A	–	A	T	C	A
KU948634	A	G	T	A	T	T	–	C	A	–	A	T	C	A
KU948635	A	G	T	A	T	T	–	C	A	–	A	T	C	A
KU948636	A	G	T	A	T	T	–	C	A	–	A	T	C	A
KU948637	A	G	T	A	T	T	–	C	A	–	A	T	C	A
KX083575	A	G	T	A	T	T	–	C	A	–	A	T	C	A
OR899267	A	G	T	A	T	T	–	C	A	–	A	T	C	A
OR899272	A	G	T	A	T	T	–	C	A	–	A	T	C	A
OR899273	A	G	T	A	T	T	–	C	A	–	A	T	C	A

∗The sequence is truncated according to the shortest sequence length.

### **3.3** Characterization of complete mitogenome (Fig. 5, Fig. 6; Table 3, Table 4)

**Fig. 5:**
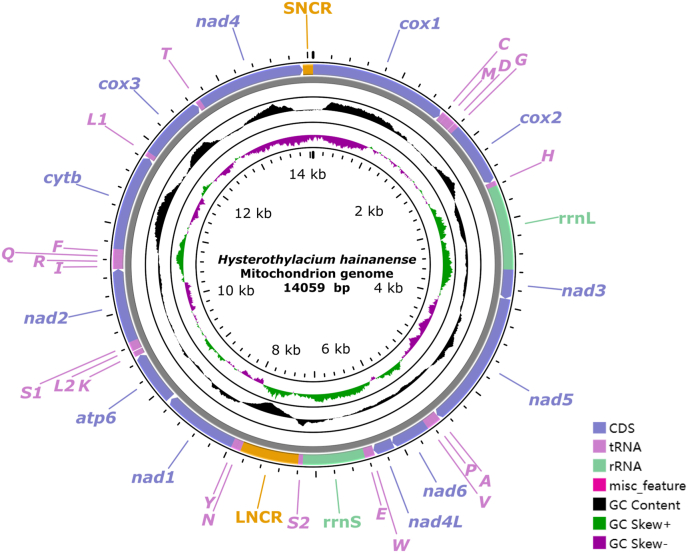
Gene map of the complete mitochondrial genome of *Hysterothylacium hainanense* sp. n. All 22 tRNA genes were nominated by the one-letter code with numbers differentiating each of the two tRNAs, serine and leucine. All genes are transcribed in the clockwise direction on the same strand. The outermost circle showed the GC content and the innermost circle showed the GC skew.

The mitogenome of *H*. *hainanense* sp. n. has 14059 bp, and contains 36 genes, including 12 protein-coding genes (PCGs) (missing *atp*8) (*cox*1–3, *cyt*b, *nad*1–6, *nad*4L and *atp*6), 22 tRNA genes and 2 rRNA genes (*rrn*L and *rrn*S), plus 2 non-coding regions (LNCR is 664 bp, between *tRNA-Ser*2 and *tRNA-Asn*; SNCR is 116 bp, between *nad*4 and *cox*1) (Fig. 5, Table 3). All genes are encoded on the same strand in the same direction. The overall A + T content in the mitogenome of *H*. *hainanense* sp. n. is 67.0%, showing a strong bias toward A + T (Table 4).

**Table 3:** Annotations and gene organization of the mitogenome of *Hysterothylacium hainanense* sp. n. The positive number in the “Gap or overlap” column indicates the length of intergenic sequence, and the negative number indicates the length (absolute number) that adjacent genes overlap (negative sign).

Gene	Type	Start (bp)	End (bp)	Length (bp)	Start Codon	Stop Codon	Anticodon	Gap or overlap
*cox*1	CDS	1	1578	1578	TTG	TAG		−1
tRNA-Cys (C)	tRNA	1578	1634	57			GCA	1
tRNA-Met (M)	tRNA	1636	1696	61			CAU	0
tRNA-Asp (D)	tRNA	1697	1755	59			GUC	4
tRNA-Gly (G)	tRNA	1760	1815	56			UCC	0
*cox*2	CDS	1816	2547	732	TTG	TAA		−1
tRNA-His (H)	tRNA	2547	2602	56			GUG	0
*rrn*L	rRNA	2603	3571	969				0
*nad*3	CDS	3572	3907	336	ATT	TAA		1
*nad*5	CDS	3909	5490	1582	ATT	T		0
tRNA-Ala (A)	tRNA	5491	5546	56			UGC	0
tRNA-Pro (P)	tRNA	5547	5604	58			UGG	0
tRNA-Val (V)	tRNA	5605	5660	56			UAC	0
*nad*6	CDS	5661	6095	435	TTG	TAA		−1
*nad*4L	CDS	6095	6328	234	ATT	TAG		0
tRNA-Trp (W)	tRNA	6329	6386	58			UCA	0
tRNA-Glu (E)	tRNA	6387	6442	56			UUC	0
*rrn*S	rRNA	6443	7147	705				0
tRNA-Ser2 (S2)	tRNA	7148	7199	52			UGA	0
LNCR	Non-coding region	7200	7863	664				0
tRNA-Asn (N)	tRNA	7864	7919	56			GUU	−1
tRNA-Tyr (Y)	tRNA	7919	7975	57			GUA	0
*nad*1	CDS	7976	8848	873	TTG	TAA		20
*atp*6	CDS	8869	9468	600	ATT	TAA		4
tRNA-Lys (K)	tRNA	9473	9534	62			UUU	9
tRNA-Leu2 (L2)	tRNA	9544	9598	55			UAA	0
tRNA-Ser1 (S1)	tRNA	9599	9650	52			UCU	0
*nad*2	CDS	9651	10494	844	TTG	T		0
tRNA-Ile (I)	tRNA	10495	10553	59			GAU	0
tRNA-Arg (R)	tRNA	10554	10608	55			ACG	0
tRNA-Gln (Q)	tRNA	10609	10663	55			UUG	0
tRNA-Phe (F)	tRNA	10664	10722	59			GAA	0
*cytb*	CDS	10723	11832	1110	ATA	TAA		0
tRNA-Leu1 (L1)	tRNA	11833	11888	56			UAG	0
*cox*3	CDS	11889	12656	768	ATT	TAG		1
tRNA-Thr (T)	tRNA	12658	12713	56			UGU	0
*nad*4	CDS	12714	13943	1230	TTG	TAA		0
SNCR	Non-coding region	13944	14059	116

**Table 4:** Base composition and nucleotide skewness of the mitogenome of *Hysterothylacium hainanense* sp. n.

Location	A%	T%	C%	G%	AT%	AT-skew	GC-skew	Total (bp)
Whole mitochondrial genome	23.7	43.3	14.4	18.6	67.0	−0.29	0.13	14059
Protein coding genes (PCGs)	20.5	44.5	15.9	19.1	65.0	−0.37	0.09	10320
1st codon	27.9	34.2	14.2	23.7	62.1	−0.10	0.25	3440
2nd codon	19.0	49.8	15.0	16.2	68.8	−0.45	0.04	3440
3rd codon	14.5	49.5	18.5	17.4	64.0	−0.55	−0.03	3440
tRNAs	31.6	37.9	11.4	19.2	69.5	−0.09	0.26	1247
rRNAs	30.8	39.9	10.9	18.5	70.7	−0.13	0.26	1674
*rrnS*	29.7	35.5	13.1	21.8	65.1	−0.09	0.25	705
*rrnL*	31.6	43.1	9.29	16.0	74.7	−0.16	0.27	969
Long non-coding region (LNCR)	39.8	41.1	8.13	11.0	80.9	−0.02	0.15	664
Short non-coding region (SNCR)	34.5	48.3	6.90	10.3	82.8	−0.17	0.20	116

**Fig. 6:**
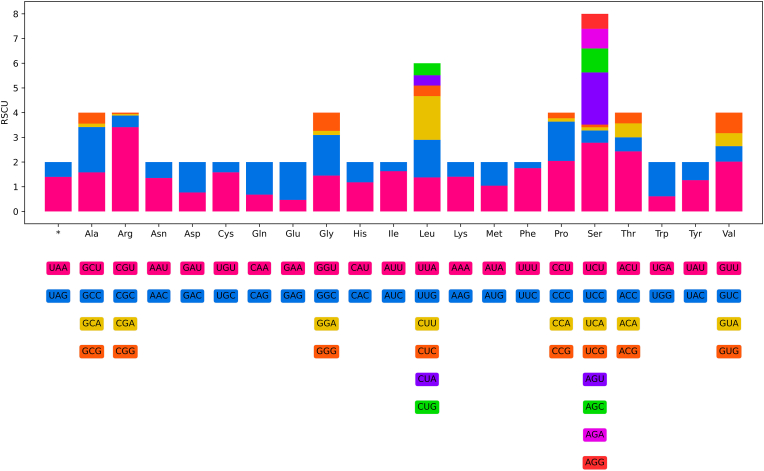
Relative synonymous codon usage (RSCU) of the mitochondrial genome of *Hysterothylacium hainanense* sp. n. The codon families (in alphabetical order) were labelled on the x-axis. Values on the top of each bar represented amino acid usage in percentage.

The 12 protein-coding genes of the mitogenome of *H*. *hainanense* sp. n. have 10320 bp (excluding termination codons), ranging in size from 234 bp (*nad*4L) to 1582 bp (*nad*5) (Fig. 5, Table 3), which encode 3430 amino acids. Among the 12 PCGs in the mitogenome of *H*. *hainanense* sp. n., TTG is the most commonly used start codon for six genes (*cox*1, *cox*2, *nad*6, *nad*1, *nad*2 and *nad*4), followed by ATT for five genes (*nad*3, *nad*5, *nad*4L, *atp*6 and *cox*3), and ATA only for *cytb*. TAA is the most commonly used termination codon for seven genes (*cox*2, *nad*3, *nad*6, *nad*1, *atp*6, *cyt*b and *nad*4), followed by TAG for three genes (*cox*1, *nad*4L and *cox*3). The remaining two genes (*nad*5 and *nad*2) are inferred to terminate with incomplete stop codon T (Table 3). The component and usages of codons in the mitogenome of *H*. *hainanense* sp. n. were shown in Fig. 6. The lengths and predicted secondary structures of 22 tRNAs of *H*. *hainanense* sp. n. were provided (Supplementary Fig. S1, Table 3).

The 36 genes arrangement in the mitogenome of *H. hainanense* sp. n. belongs to the GA3 type of Nematoda, according to the following order: *cox*1, *tRNA-Cys*, *tRNA-Met*, *tRNA-Asp*, *tRNA-Gly*, *cox*2, *tRNA-His*, *rrn*L, *nad*3, *nad*5, *tRNA-Ala*, *tRNA-Pro*, *tRNA-Val*, *nad*6, *nad*4L, *tRNA-Trp*, *tRNA-Glu*, *rrn*S, *tRNA-Ser*2, *tRNA-Asn*, *tRNA-Tyr*, *nad*1, *atp*6, *tRNA-Lys*, *tRNA-Leu*2, *tRNA-Ser*1, *nad*2, *tRNA-Ile*, *tRNA-Arg*, *tRNA-Gln*, *tRNA-Phe*, *cyt*b, *tRNA-Leu*1, *cox*3, *tRNA-Thr*, *nad*4 (Fig. 5).

## 4 Discussion

In the genus *Hysterothylacium*, *H*. *hainanense* sp. n. with narrow lateral alae, short intestinal caecum (not over 30.0% of oesophageal length) and long ventricular appendix (about 2–6 times longer than intestinal caecum), equal spicules (0.30–0.90 mm long), and tail tip covered by numerous spines or nodular protuberances in both sexes, is similar to the following species: *H. fabri* (Rudolphi, 1819), *H. seriolae* (Yamaguti, 1941), *H. fortalezae* (Klein, 1973), *H. scomberoidei* (Bruce and Cannon, 1989), *H. chrysostomi* Bruce (1990), *H. physiculi* (Moravec and Nagasawa, 2000), *H. winteri* (Torres and Soto, 2004), *H. reliquens* (Norris and Overstreet, 1975), *H. perezi* (Gopar-Merino et al., 2005*)*, *H*. *arnoglossi* (Petter and Maillard, 1987) and *H. australe* (Shamsi, 2017) (Yamaguti, 1941; Klein, 1973; Norris and Overstreet, 1975; Deardorff and Overstreet, 1980; Petter and Maillard, 1987; Bruce and Cannon, 1989; Bruce, 1990; Moravec and Nagasawa, 2000; Torres and Soto, 2004; Gopar-Merino et al., 2005; Shamsi, 2017; Zhao et al., 2017).

*Hysterothylacium hainanense* sp. n. differs from *H. reliquens* by the different morphology of lips and relatively shorter spicules (spicules representing 2.30–3.20% of body length in the new species *vs* 5.40–8.40% of body length in *H. reliquens*). The new species can be easily distinguished from *H. chrysostomi* and *H. australe* by distinctly less postcloacal papillae (4–5 pairs in the former *vs* 9–12 pairs in the latter two species). *Hysterothylacium hainanense* sp. n. is different from *H. winteri* by distinctly shorter oesophagus in male (oesophagus 1.02–1.73 mm long, representing 4.70–10.8% of body length in the new species *vs* 3.90–6.60 mm, representing 12.9–21.3% of body length in *H. winteri*). The new species also differs from *H. physiculi* by the distinctly shorter oesophagus and tail in the female (oesophagus 1.07–1.66 mm long, tail 0.13–0.35 mm in *H*. *hainanense vs* oesophagus 2.83–3.87 mm long, tail 0.87–1.31 mm in *H. physiculi*). *Hysterothylacium hainanense* sp. n. can be differentiated from *H. arnoglossi* by slightly shorter spicules (0.38–0.80 mm long, representing 2.30–3.20 % of body length in the new species *vs* 0.84–1.30 mm long, representing 4.10–6.20 % of body length in the latter).

*Hysterothylacium fortalezae* and *H. scomberoidei* with lateral alae starting from the base of ventro-lateral lips and remarkably expanded at cervical region, are different from the new species (*vs* lateral alae extending from some distance from base of ventro-lateral lips and very narrow in cervical region in *H*. *hainanense*). *Hysterothylacium perezi* has particular medioventral postcloacal pad and 39–43 pairs of precloacal papillae, which can be easily distinguished from *H*. *hainanense* sp. n. (*vs* lacking of postcloacal pad and presence of 25–27 pairs of precloacal papillae). Yamaguti (1941) reported *H. seriolae* from *Seriola quinqueradiata* Temminck & Schlegel (Carangiformes: Carangidae) off the coast of Japan (Yamaguti, 1941). However, the original description of this species is very simple with only one illustration. Consequently, we requested the loan of the type series of *H. seriolae*, deposited in the Meguro Parasitological Museum Tokyo, Japan. The curator of the collection, Dr. Takeshi Iwaki, provided some important photomicrographs of the type specimens of *H. seriolae*, which clearly showed the morphology of lips and tail of *H. seriolae* differing from that of the new species. Additionally, *H. seriolae* with 19–25 pairs of precloacal papillae, 6 pairs of postcloacal papillae in male and oesophageal length 1.90–2.50 mm in female, is different from that of the new species (presence of 25–27 pairs of precloacal papillae and 4–5 pairs of postcloacal papillae, oesophageal length 1.07–1.66 mm in female).

*Hysterothylacium fabri* is a common ascaridoid species reported from various fishes in the Mediterranean Sea (Bruce et al., 1994; Petter and Maillard, 1987, 1988; Petter and Radujkovic, 1989; Tedesco et al., 2018). The morphological characters and genetic data of *H. fabri* were reported by some studies (Petter and Maillard, 1987, 1988; Petter and Radujkovic, 1986, 1989; Martín-Sánchez et al., 2003; Li et al., 2008a, 2018; Pekmezci et al., 2014; Tedesco et al., 2018). According to the previous studies, the adults of *H*. *fabri* exhibited a broad range of morphological variability in the lengths of body, oesophagus and spicules, the ratio of intestinal caecum to ventricular appendix and the number and arrangement of caudal papillae (Petter and Maillard, 1987; Petter and Radujkovic, 1989; Tedesco et al., 2018). The previous molecular analysis considered that *H. fabri* parasitizes the Mediterranean fishes representing a complex species comprising at least three sibling species with low host specificity (Martín-Sánchez et al., 2003). The present new species and *H. fabri* are rather morphologically similar, and both parasitize uranoscopid fishes as definitive hosts, but *Hysterothylacium hainanense* sp. n. differs from *H. fabri* by having slightly smaller body (13.8–28.0 mm long in the new species *vs* 25.6–72.7 mm long in *H. fabri*), shorter spicules (0.37–0.80 mm long in *H. hainanense* sp. n. *vs* 0.65–1.42 mm in the latter) and different number and arrangement of caudal papillae (precloacal 25–27 pairs, paracloacal absent and postcloacal 4–5 pairs in the former *vs* precloacal 21 pairs in *H. fabri*, paracloacal 1–2 pairs and postcloacal 5–9 pairs in *H. fabri*) in male (Petter and Maillard, 1987; Petter and Radujkovic, 1986, 1989; Tedesco et al., 2018). Moreover, the 10.13039/100018231ASAP and BI results both supported that *H*. *hainanense* sp. n. and *H. fabri* represent two distinct species. Pairwise comparison also revealed the presence of some loci of nucleotide polymorphisms in the ITS region, which possibly represent fixed divergences between *H*. *hainanense* sp. n. and *H. fabri*, which are accordant with the ASAP and BI results. Additionally, we did not find remarkable morphological variability between the specimens of *H*. *hainanense* sp. n. from *U. tosae* and *U. japonicus*, which are accordant with the results of pairwise comparison (no nucleotide difference detected in the ITS region between the different individuals of this new species from the two different hosts).

In the superfamily Ascaridoidea, there have been 33 species with their mitogenomic data sequenced, but most of these mitochondrial genomic data available for ascaridoids belong to the families Anisakidae, Ascarididae and Toxocaridae with veterinary, medical and economic importance (Supplementary Table S1) (Liu et al., 2015; Xie et al., 2011a, Xie et al., 2011b, 2022; Jabbar et al., 2014; Li et al., 2008b; Gao et al., 2019, 2024; Gu et al., 2023; Chen et al., 2021; Han et al., 2022; Mohandas et al., 2014; Park et al., 2011; Wolstenholme et al., 1994; Yamada et al., 2017; Zhao et al., 2018, 2021). There is no raphidascaridid or *Hysterothylacium* species with the mitochondrial genomic data reported so far. The complete mitochondrial genome of *H*. *hainanense* sp. n. represents the first mitogenomic data for the genus *Hysterothylacium*, and also for the family Raphidascarididae. The mitogenome of *H. hainanense* sp. n. is 14059 bp in length, which is slightly larger than that of all of the known mitogenomes of the anisakid species and the single representative of Heterocheilidae (*Ortleppascaris sinensis*), but is similar to most of the mitogenomes of ascaridid and toxocarid nematodes (Supplementary Table S1). It is interesting that the overall A + T content in the mitogenome of *H. hainanense* sp. n. (67.0%) is lower than that of all of the ascaridoid mitogenomes reported so far (Supplementary Table S1). The 36 gene arrangement in the mitogenome of *H. hainanense* sp. n. belong to the GA3 type, a typical gene order of ascaridoid mitogenomes. The gene arrangement order seems to be highly conservative within ascaridoid mitogenomes, but is different from that of the superfamilies Heterakoidae (GA1 type) and Seuratoidea (GA6 type and GA 54 type) in Ascaridida. The present study enriched the mitogenomic data of ascaridoid nematodes, and revealed the patterns of mitogenomic evolution of the Raphidascarididae and *Hysterothylacium* for the first time.

## CRediT authorship contribution statement

**Hui-Xia Chen:** Writing – original draft, Data curation, Formal analysis, Methodology. **Hai-Xia Qiao:** Data curation, Formal analysis, Methodology. **Wen-Ting Zhao:** Data curation, Formal analysis, Methodology. **Xiao-Hong Gu:** Data curation, Formal analysis, Methodology. **Liang Li:** Writing – original draft, Data curation, Formal analysis, Project administration, Supervision, Writing – review & editing.

## Ethical approval

This study was conducted under the protocol of Hebei Normal University (protocol number LLSC2024090). All applicable institutional, national and international guidelines for the care and use of animals were followed.

## Data availability

The ITS and mitogenomic sequence data of *H. hainanense* sp. n. generated in this study were deposited in the GenBank database (under the accession numbers PQ738935‒PQ738939, PQ740960).

## Funding

This study was supported by the 10.13039/501100001809National Natural Science Foundation of China (Grant No. 32170442), and the 10.13039/501100003481Key Development Foundation of Hebei Normal University (L2024ZD17) for Dr. Liang Li.

## Conflict of interest

The authors declare that they have no competing interests.

## Figures and Tables

**Figure:**
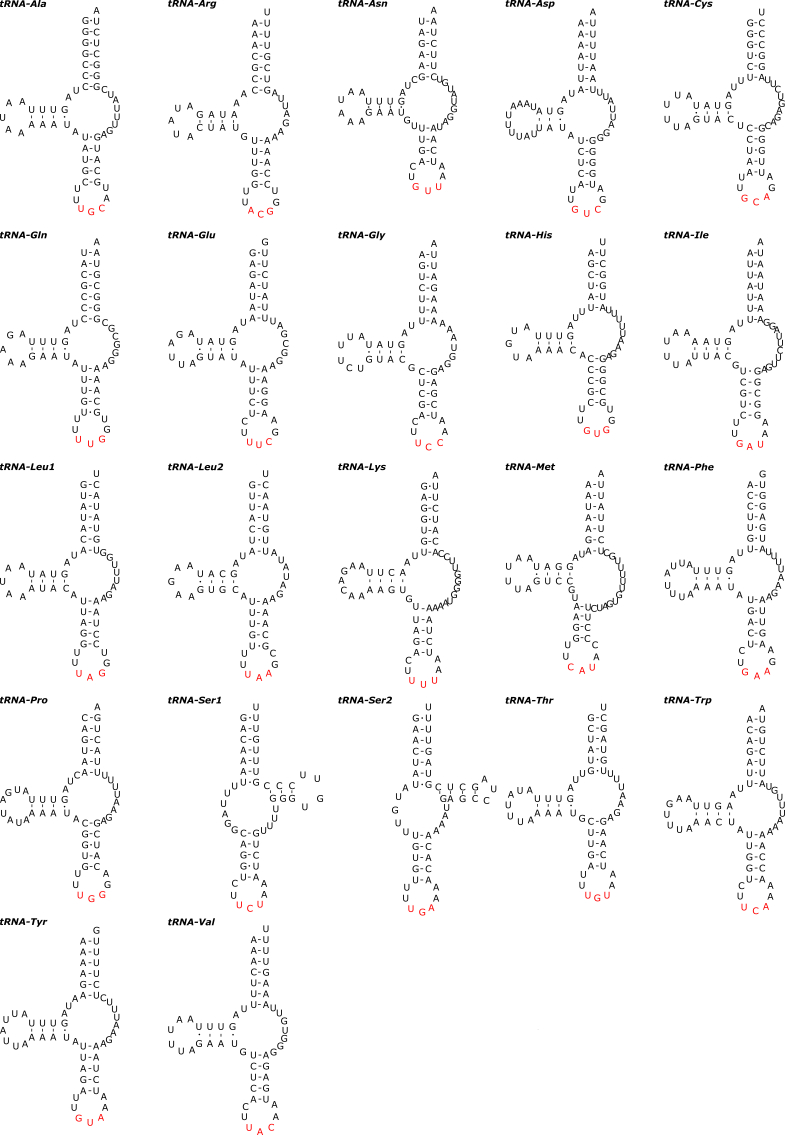
Supplementary Fig. S1Inferred secondary structures of 22 tRNAs in the mitogenome of *Hysterothylacium hainanense* sp. n. Lines between bases indicated Watson–Crick bonds, dots indicated GU bonds and bases in red represent anticodons. tRNA, Transfer RNA.
