# Supplementary material for: Integrative evidence reveals a new species of Hysterothylacium (Nematoda: Ascaridoidea), with the characterization of its complete mitochondrial genome
Source: Int J Parasitol Parasites Wildl. 2025 Feb 2;26:101042. doi: 10.1016/j.ijppaw.2025.101042 (PMC11848143; doi:10.1016/j.ijppaw.2025.101042)
Supplement: Multimedia component 1 [file mmc1.docx]

**Supplementary Table S1.** Detailed information of mitogenomes of ascaridoid species reported so far.

| Species | GenBank ID | Length (bp) | AT% | Non coding region | References |
| --- | --- | --- | --- | --- | --- |
|  |  |  |  |  |  |
| **Anisakidae** |  |  |  |  |  |
| *Anisakis berlandi* | NC_026023 | 13915 | 71.3 | 2 | Unpublished |
| *Anisakis pegreffii* | NC_034329 | 14002 | 71.4 | 2 | Yamada et al. (2017) |
| *Anisakis simplex* | KU899549 | 13938 | 71.3 | 3 | Unpublished |
| *Contracaecum ogmorhini* | NC_031647 | 14019 | 71.4 | 2 | Unpublished |
| *Contracaecum osculatum* | NC_024037 | 13823 | 70.2 | 4 | Mohandas et al. (2014) |
| *Contracaecum rudolphii* | NC_014870 | 14022 | 70.5 | 2 | Unpublished |
| *Pseudoterranova azarasi* | NC_027163 | 13954 | 70.7 | 2 | Liu et al. (2015) |
| *Pseudoterranova bulbosa* | NC_031643 | 13957 | 71.2 | 2 | Unpublished |
| *Pseudoterranova cattani* | NC_031644 | 13950 | 71.1 | 2 | Unpublished |
| *Pseudoterranova decipiens* | NC_031645 | 13962 | 71.0 | 2 | Unpublished |
| *Pseudoterranova krabbei* | NC_031646 | 13948 | 70.4 | 2 | Unpublished |
| **Ascarididae** |  |  |  |  |  |
| *Ascaris lumbricoides* | NC_016198 | 14281 | 71.8 | 2 | Park et al. (2011) |
| *Ascaris ovis* | MT993838 | 14205 | 72.0 | 2 | Chen et al. (2021) |
| *Ascaris suum* | NC_001327 | 14284 | 72.0 | 2 | Wolstenholme et al. (1994) |
| *Baylisascaris ailuri* | NC_015925 | 14657 | 69.5 | 2 | Xie et al. (2011b) |
| *Baylisascaris procyonis* | JF951366 | 14781 | 70.5 | 2 | Xie et al. (2011a) |
| *Baylisascaris schroederi* | HQ671081 | 14778 | 68.6 | 2 | Xie et al. (2011b) |
| *Baylisascaris transfuga* | HQ671079 | 14898 | 69.5 | 2 | Xie et al. (2011b) |
| *Parascaris equorum* | NC_036427 | 13899 | 70.3 | 2 | Gao et al. (2019) |
| *Parascaris univalens* | NC_024884 | 13920 | 70.6 | 2 | Jabbar et al. (2014) |
| *Toxascaris leonina* | MK516267 | 14685 | 71.1 | 2 | Unpublished |
| *Ophidascaris baylisi* | MW880927 | 14784 | 70.0 | 2 | Zhao et al. (2021) |
| *Ophidascaris wangi* | MK106624 | 14660 | 69.2 | 2 | Unpublished |
| *Ophidascaris* sp. | CNA0050675 | 14641 | 70.2 | 2 | Han et al. (2022) |
| **Heterocheilidae** |  |  |  |  |  |
| *Ortleppascaris sinensis* | KU950438 | 13828 | 74.0 | 1 | Zhao et al. (2018) |
| **Raphidascarididae** |  |  |  |  |  |
| *Hysterothylacium hainanense* sp. n. | PQ740960 | 14059 | 67.0 | 2 | Present study |
| **Toxocaridae** |  |  |  |  |  |
| *Toxocara apodemi* | OR241493 | 14303 | 68.4 | 2 | Gao et al. (2024) |
| *Toxocara canis* | NC_010690 | 14322 | 68.6 | 2 | Li et al. (2008b) |
| *Toxocara cati* | NC_010773 | 14029 | 70.0 | 2 | Li et al. (2008b) |
| *Toxocara malaysiensis* | NC_010527 | 14266 | 68.9 | 2 | Li et al. (2008b) |
| *Toxocara vitulorum* | NC_070176 | 15045 | 70.0 | 2 | Xie et al. (2022) |
| *Porrocaecum moraveci* | OQ863051 | 14517 | 70.0 | 3 | Gu et al. (2023) |
| *Porrocaecum reticulatum* | OQ863050 | 14210 | 67.2 | 2 | Gu et al. (2023) |
| *Porrocaecum* sp. | CNA0050678 | 14568 | 71.4 | 2 | Han et al. (2022) |
